# Anhydrous Alum Inhibits α-MSH-Induced Melanogenesis by Down-Regulating MITF via Dual Modulation of CREB and ERK

**DOI:** 10.3390/ijms241914662

**Published:** 2023-09-28

**Authors:** Kyu-Ree In, Mi Ae Kang, Su Dong Kim, Jinho Shin, Sung Un Kang, Tae Jun Park, Seung-Joo Kim, Jong-Soo Lee

**Affiliations:** 1 Department of Life Sciences, College of Natural Sciences, Ajou University, Suwon 16499, Republic of Korea; 2 Research Institute of Basic Sciences, Ajou University, Suwon 16499, Republic of Korea; 3 Graduate School of Clinical Pharmacy and Pharmaceutics, Ajou University, Suwon 16499, Republic of Korea; 4 Department of Chemistry, College of Natural Sciences, Ajou University, Suwon 16499, Republic of Korea; 5 Department of Otolaryngology, School of Medicine, Ajou University, Suwon 16499, Republic of Korea; 6 Department of Biomedical Science, The Graduate School, Ajou University, Suwon 16499, Republic of Korea; 7 Department of Biochemistry and Molecular Biology, Ajou University School of Medicine, Suwon 16499, Republic of Korea

**Keywords:** anhydrous alum, melanogenesis, MITF, CREB, ERK

## Abstract

Melanogenesis, the intricate process of melanin synthesis, is central to skin pigmentation and photoprotection and is regulated by various signaling pathways and transcription factors. To develop potential skin-whitening agents, we used B16F1 melanoma cells to investigate the inhibitory effects of anhydrous alum on melanogenesis and its underlying molecular mechanisms. Anhydrous alum (KAl(SO_4_)_2_) with high purity (>99%), which is generated through the heat-treatment of hydrated alum (KAl(SO_4_)_2_·12H_2_O) at 400 °C, potentiates a significant reduction in melanin content without cytotoxicity. Anhydrous alum downregulates the master regulator of melanogenesis, microphthalmia-associated transcription factor (MITF), which targets key genes involved in melanogenesis, thereby inhibiting α-melanocyte-stimulating hormone (α-MSH)-induced melanogenesis. Phosphorylation of the cAMP response element-binding protein, which acts as a co-activator of MITF gene expression, is attenuated by anhydrous alum, resulting in compromised MITF transcription. Notably, anhydrous alum promoted extracellular signal-regulated kinase phosphorylation, leading to the impaired nuclear localization of MITF. Overall, these results demonstrated the generation and mode of action of anhydrous alum in B16F1 cells, which constitutes a promising option for cosmetic or therapeutic use.

## 1. Introduction

Melanin determines skin color. Additionally, melanin protects against damaging agents by absorbing oxidative free radicals generated intracellularly or environmentally and safeguards the skin from ionizing radiation, including UV radiation [1].

However, the overproduction of melanin causes a variety of hyperpigmentation disorders, such as melasma, chloasma, acanthosis nigricans, freckles, solar lentigo, and melanoma [2,3,4], which impact the quality of life and may require cosmetic intervention. In addition, melanin and its highly reactive intermediates have a positive and negative role in melanoma development, progression, and further therapy [5]. Therefore, the discovery and development of melanogenesis inhibitors are crucial [6,7,8].

Melanin is the primary cause of skin pigmentation and is produced by pigment-producing melanocytes during melanogenesis [9]. Melanin is formed through a combination of enzymatic and non-enzymatic reactions, which are regulated by various signaling pathways [10]. The initiation of melanogenesis by the key enzyme tyrosinase is the only rate-limiting step in the melanin synthesis pathway because all other steps proceed spontaneously under normal physiological conditions [11]. In addition to tyrosinase, tyrosinase-related proteins 1 and 2 (TRP1 and TRP2, respectively) participate in melanin synthesis [12]. Tyrosinase is essential for melanogenesis and is crucial for the ensuing spontaneous reactions. Therefore, tyrosinase activity is the crucial first control point of melanogenesis. A reduction in tyrosinase activity has been targeted for the prevention or treatment of hyperpigmentation-related disorders in the pharmaceutical and cosmetic industry [13]. 

Melanogenesis is regulated at multiple levels, including the migration of melanocytes into target sites, formation of melanosomes in melanocytes, and gene expression of melanogenesis-related enzymes, including tyrosinase and TRP1/2. In melanocytes, the gene expression of melanogenesis enzymes is regulated via signaling pathways initiated by a variety of ligands, including hormones (alpha-melanocyte-stimulating hormone (α-MSH)) and growth factors (SCF and Wnt), in response to UV radiation or stress [14]. These signaling pathways involve the transcription factor microphthalmia-associated transcription factor (MITF), which functions as a master regulator of melanogenesis via regulating the expression of tyrosinase and TRP1/2 [13]. Accordingly, the transcriptional activity of melanogenesis is the key regulator of MITF. The up- and downregulation of MITF can facilitate and suppress melanogenesis by activating and inactivating the transcription of related enzyme genes, respectively.

Melanogenesis is regulated by proopiomelanocortin (POMC) and its peptides including melanocyte-stimulating hormone (α-MSH), and also other melanogenic neuropeptides that are produced in the skin [15]. UVB can upregulate α-MSH receptor (MC1R) expression, POMC expression, and the production of POMC-peptides, which regulates skin pigmentation in response to UV irradiation [16]. Particularly, α-MSH stimulates melanogenesis via a cyclic AMP (cAMP)-dependent pathway. Once α-MSH binds to its membrane receptor in melanocytes, adenylate cyclase is activated to produce cAMP, which subsequently activates protein kinase A (PKA). PKA phosphorylates the cAMP response element-binding protein (CREB) to function as a coactivator of MITF transcription [17]. MITF levels are increased via the α-MSH-cAMP-CREB pathway, which activates the relevant melanogenesis enzyme genes and promotes melanogenesis [18]. Additionally, the Wnt signal pathway upregulates the expression of the MITF gene via inhibiting the degradation of β-catenin proteins [19]. In contrast to the upregulation of MITF gene expression via the α-MSH and Wnt pathways, the activation of the extracellular signal-regulated kinase (ERK) pathway promotes the phosphorylation and degradation of MITF, thereby inhibiting melanogenesis [13]. In combination, these signaling pathways control MITF levels, affecting its nuclear level and transcriptional activity. Finally, these intracellular pathways modulate the level of the key melanogenesis enzyme, tyrosinase [13,17,18], implying that the pathways function upstream of tyrosinase transcription by MITF. Therefore, understanding the complex interplay between these signaling pathways and the transcription factor MITF for melanogenesis is essential for developing novel cosmetics and therapeutics for pigmentation disorders and melanoma. In addition to targeting tyrosinase, melanogenesis inhibition could be accomplished by targeting these pathways.

Tyrosinase inhibitors are widely used as therapeutic agents for treating melanin hyperpigmentation and as cosmetic materials for skin whitening after sunburn. Various tyrosinase inhibitors, including kojic acid, hydroquinone, and arbutin, have been developed [6,8,20,21,22]. Tyrosinase inhibitors are also used in the food industry because tyrosinase generates a brownish pigment when its oxidative and reactive product, dopamine quinone, reacts with amino acids and proteins [23]. However, these substances have limitations and side effects including low effectiveness, reliability, high toxicity, poor permeability, and a lack of stability [24,25,26,27]. Therefore, effective, safe, and biocompatible skin-whitening agents that can target tyrosinase activity and related signaling pathways to control human skin hyperpigmentation must be developed [28]. 

Hydrated potassium aluminum sulfate (KAl(SO_4_)_2_·12H_2_O), also known as potassium alum, potash alum, or alum, is a chemical compound belonging to the generic class of double sulfate salts [29,30,31]. KAl(SO_4_)_2_·12H_2_O converts to the KAl(SO_4_)_2_ via dehydration at approximately 240 °C [32]. Because hydrated alum is generally recognized as a safe substance, it has long been used as a food additive and component in various cosmetic formulations, such as antiperspirants, deodorants, and skin care products [33]. Hydrated alum is a traditional Chinese medicine with astringent and antibacterial properties. This material has been used as a styptic substance in traditional medicine to treat open wounds and sores. In modern medicine, anhydrous and hydrated alum are frequently used as major ingredients in external ointments to cure eczema, pruritus, and otitis media. Anhydrous alum can be prepared by performing a typical heat treatment, called “roasting”, on the hydrated alum. Although both hydrated and roasted (anhydrous) alum have been used in various external medicines and cosmetics, the dermatological effects of these two substances have not been compared. Only the antioxidant and anti-inflammatory effects of hydrated and roasted (anhydrous) alum have been compared recently [33]. 

In the present study, we used anhydrous alum to develop a promising skin-whitening agent for treating pigmentation-related diseases. The structural features of anhydrous alum were compared with those of hydrated alum. The cellular toxicity and inhibition of melanin biosynthesis in melanoma B16F1 cells were further evaluated. Finally, the mechanism of action of anhydrous alum that inhibited melanogenesis was elucidated.

## 2. Results

### 2.1. Structural Characterization of Pure Anhydrous Alum

**Figure 1:**
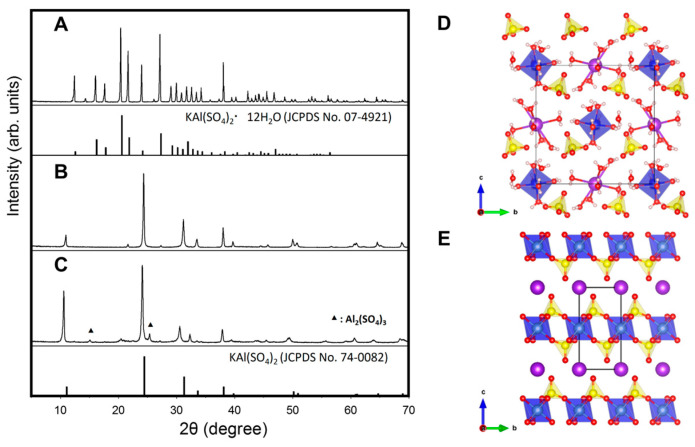
X-ray diffraction (XRD) patterns for (**A**) hydrated alum (H-alum), (**B**) anhydrous alum (A-alum-1), and (**C**) commercial anhydrous alum (A-alum-2), and the crystal structures including unit cell of (**D**) KAl(SO_4_)_2_·12H_2_O and (**E**) KAl(SO_4_)_2_. In the crystal structures, AlO_6_ octahedron and SO_4_ tetrahedron are shown in blue and yellow, respectively. K, O and H atoms are represented by large purple spheres and small white spheres, respectively.

The X-ray diffraction (XRD) patterns of the hydrated alum (H-alum), which comprised anhydrous alum (A-alum-1) prepared by roasting the H-alum at 400 °C for 4 h, and a commercial anhydrous alum (A-alum-2) are shown in Figure 1A–C. The H-alum was identified as KAl(SO_4_)_2_·12H_2_O (JCPDS No. 07-4921). In the crystal structure of hydrated alum (Figure 1D), half of the water molecules in a unit cell are coordinated to Al^3+^ and the other half to K^+^ cations, forming [Al(H_2_O)_6_]^3+^ and [K(H_2_O)_6_]^+^ octahedra. All water molecules are linked to the SO_4_ groups via hydrogen bonds [29]. A-alum-1 appears to be a pure phase of anhydrous alum, KAl(SO_4_)_2_ (JCPDS No. 74-0082), with a hexagonal unit cell with a = 4.716(5) Å, c = 7.997(6) Å, γ = 120°, and V = 154.07 (1) Å^3^. In the crystal structure of KAl(SO_4_)_2_ (Figure 1E), the Al^3+^ was bonded to six O^2-^ atoms to form AlO_6_ octahedra that shared corners with six equivalent SO_4_ tetrahedra, resulting in two-dimensional [Al(SO_4_)_2_]^−^ slabs. K^+^ ions are located in the interlayer space between the two [Al(SO_4_)_2_]^−^ slabs and interact with the terminal oxygen atoms of the SO_4_ group [29]. A-alum-2 is composed of a predominant KAl(SO_4_)_2_ phase and a secondary aluminum sulfate (Al_2_(SO_4_)_3_, in contrast to the single KAl(SO_4_)_2_ phase of A-alum-1. The content of Al_2_(SO_4_)_3_ in A-alum-2 was estimated to be approximately 7 ± 1% by XRD profile analysis.

**Figure 2:**
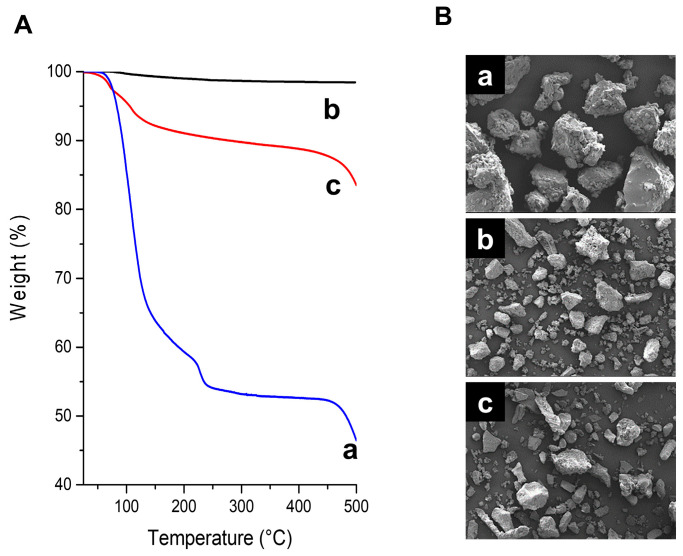
Thermogravimetric analyses and morphological Scanning Electron Microscope (SEM) images of H-alum, A-alum-1, and A-alum-2. (**A**) Thermogravimetric analysis under air atmosphere. (**B**) SEM images of alums. a: H-alum; b: A-alum-1; c: A-alum-2.

The thermal behaviors of the three alum samples over the temperature range 25–500 °C were monitored using thermogravimetric (TG) measurement. As shown in Figure 2A, H-alum underwent stepwise weight loss during the heating process. The first weight loss in the temperature range of 100–220 °C was attributed to the removal of water molecules from the KAl(SO_4_)_2_·12H_2_O lattice. The weight loss (~46.7%) observed at this stage was consistent with the theoretical weight loss (45.6%) calculated using the reaction KAl(SO_4_)_2_·12H_2_O → KAl(SO_4_)_2_ + 12H_2_O. The second weight loss occurring above 480 °C is attributed to the decomposition of anhydrous salts involving desulfurization [32]. The pure anhydrous alum (A-alum-1) exhibited a slight weight loss (<3%) corresponding to the removal of adsorbed moisture, and no further weight loss was observed during the heating process to 500 °C. However, the A-alum-2 showed a substantial amount of weight loss (~10%), which might be due to the liberation of additional water molecules from the surface of the hygroscopic Al_2_(SO_4_)_3_.

The morphologies of the alum samples are shown in Figure 2B. The grains exhibited irregular shapes with rough surfaces composed of numerous satellite particles. The particle sizes of A-alum-1 and A-alum-2 are moderately smaller than those of H-alum.

### 2.2. Pure Anhydrous Alum Inhibited Melanogenesis with Little Cytotoxicity

**Figure 3:**
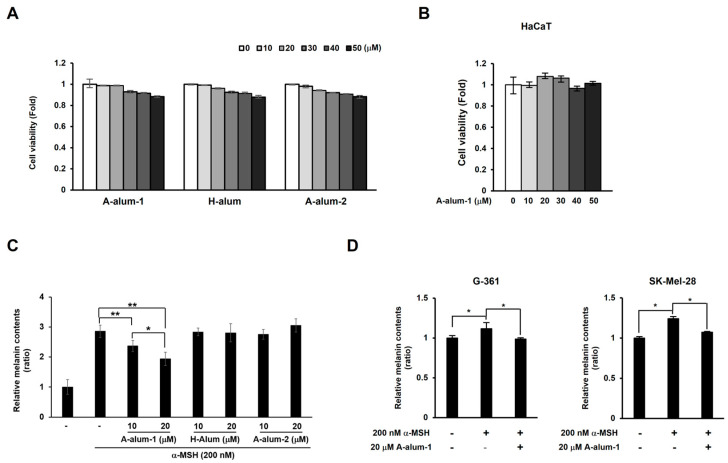
Effects of A-alum-1, H-alum, and A-alum-2 on the cell viability and melanin contents in melanoma and keratinocyte cells. (**A**) Cell viability was determined using resazurin reduction assay following treatments with A-alum-1, H-alum, or A-alum-2 at various concentrations (10–50 μM) in the B16F1 cells. (**B**) The human keratinocyte HaCaT cells were treated with indicated concentrations of A-alum-1 for 48 h and cell viability was determined. Values are mean ± SD from six experiments. The viability of the untreated cells was arbitrarily set to one. The amount of melanin was measured in B16F1 cells (**C**) pretreated with A-alum-1, H-alum, or A-alum-2 (10 or 20 μM), and G-361 and SK-Mel-28 cells (**D**) pretreated with 20 μM A-alum-1 for 1 h prior to treatment with α-MSH (200 nM) for 72 h. The melanin content in the untreated cells was arbitrarily set to one. Values are mean ± SD from six experiments; * *p* < 0.05, ** *p* < 0.01.

To evaluate the cytotoxic effects of the pure anhydrous alum (A-alum-1), the cell viability of B16F1 cells treated with A-alum-1, A-alum-2, and H-alum at various concentrations ranging between 10 and 50 μM for 48 h was assessed by using a resazurin reduction assay. Up to 20 μM, A-alum-1 did not affect cell viability and exhibited subtle effects on the viability at approximately 30–50 μM in B16F1 cells. Therefore, A-alum-1 concentrations of approximately 10–20 μM were determined for further experiments. Similar to A-alum-1, both H-alum and A-alum-2 demonstrated minimal effects on cell viability at all tested doses (Figure 3A). A-alum-1 also did not affect the viability of human HaCaT keratinocytes (Figure 3B). These results suggested that A-alum-1 exhibited little cytotoxicity.

To investigate the inhibitory effect of A-alum-1 on melanogenesis, we measured melanin content in α-MSH-induced B16F1 cells. A-alum-1 was found to significantly reduce the α-MSH-induced melanin quantity in a dose-dependent manner (Figure 3C). As shown in Figure 3C, the α-MSH-induced melanin content was enhanced by approximately 2.85 ± 0.21-fold compared to that in the untreated cells. A-alum-1 decreased the α-MSH induced melanin content to 83.2 ± 6.7% (10 μM) or 68.1 ± 7.7% (20 μM). In contrast to the inhibition of α-MSH-induced melanin synthesis by A-alum-1, neither H-alum nor A-alum-2 exhibited hypopigmentation activity (Figure 3C). To address whether A-alum-1 consistently reduced the α-MSH-induced melanin amount, we extended the application to human melanoma cell lines. We found that A-alum-1 treatment suppressed α-MSH-induced melanin synthesis in human G-361 and SK-Mel-28 cells (Figure 3D). Thus, these data indicated that the pure anhydrous A-alum-1 has a stronger inhibitory effect on the α-MSH-induced melanin synthesis, in contrast to the hydrated H-alum and commercial anhydrous A-alum-2 lacking melanogenesis inhibition activity in three tested melanoma cell lines.

### 2.3. A-Alum-1 Suppresses Tyrosinase Gene Expression by Reducing MITF Expression in α-MSH-Induced B16F1 Cells

**Figure 4:**
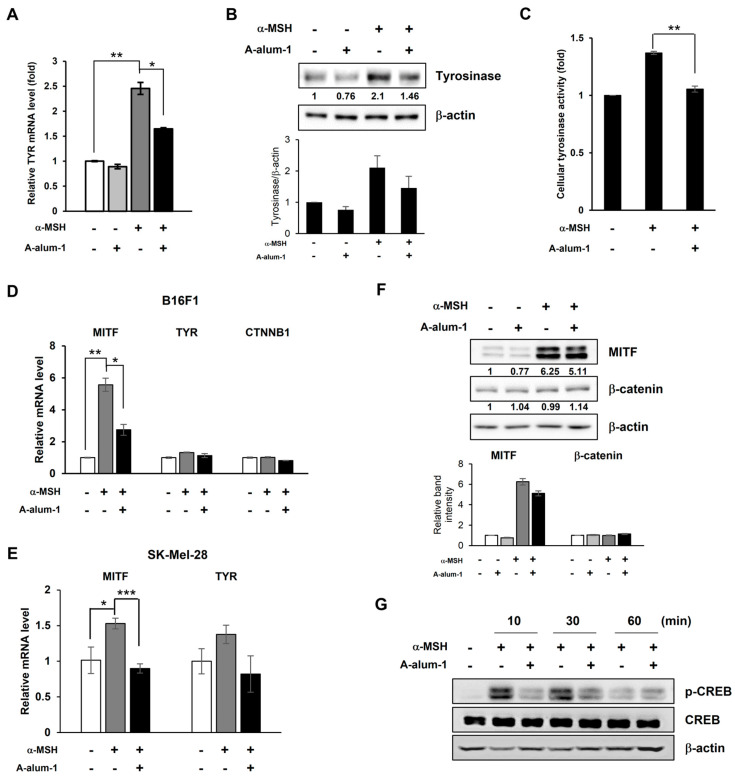
Inhibitory effects of A-alum-1 on the levels of mRNAs (**A**,**D**,**E**) or proteins (**B**,**F**,**G**) related to melanogenesis in α-MSH-stimulated B16F1 (**A**–**D**,**F**,**G**) and SK-Mel-28 (**E**) cells were exposed to 200 nM α-MSH in the absence (-) or presence of 20 µM A-alum-1 for 24 h (**A**), 48 h (**B**), 2 h (**D**,**E**), or 4 h (**F**). The expression levels of MITF, β-catenin, and tyrosinase were measured using qRT-PCR (**A**,**D**,**E**) and Western blotting (**B**,**F**). (**C**) B16F1 cells treated with 200 nM α-MSH in the presence or absence of 20 μM A-alum-1 for 72 h. The cells were lysed and measured cellular tyrosinase activity. (**G**) The phosphorylation level of CREB was determined at the indicated time using Western blotting. β-actin was used as the loading control. Each Western blot was representative of three different experiments. Relative intensities of the indicated proteins are shown at the bottom of the Western blot and in the histogram. * *p* < 0.05, ** *p* < 0.01, *** *p* < 0.001.

To elucidate the molecular mechanisms underlying the inhibition of melanogenesis by A-alam-1, we first investigated the effect of A-alum-1 on the expression levels of mRNAs corresponding to TYR using quantitative reverse transcription polymerase chain reaction (qRT-PCR), and its protein products via Western blotting in α-MSH-induced B16F1 cells. As shown in Figure 4A, the α-MSH-induced level of tyrosinase mRNA was elevated to approximately 2.5-fold in comparison with that seen in the absence of α-MSH (control). The α-MSH-induced tyrosinase mRNA expression was reduced to approximately 67.3% by A-alum1-1 compared to that observed following treatment with α-MSH alone (Figure 4A). Similarly, A-alum-1 also effectively reduced the tyrosinase protein expression level in α-MSH-induced cells (Figure 4B). Additionally, α-MSH-induced cellular tyrosinase activity was consistently decreased by A-alum-1 treatment (Figure 4C). These results suggest that the inhibition of melanogenesis by A-alum-1 involves a reduction in the expression and activity of the key melanogenesis enzyme tyrosinase in B16F1 cells.

To understand the mechanism of A-alum-1-mediated tyrosinase suppression in B16F1 cells, we next studied whether A-alum-1 downregulates the expression of the master melanogenesis transcription factor MITF, which activates key melanogenesis enzyme genes such as tyrosinase, thereby inhibiting melanogenesis. Although we observed a subtle induction of MITF mRNA (~1.4-fold compared to non-treatment, control) and protein expression after 24 h of α-MSH treatment (Supplementary Figure S1), MITF protein expression was previously found to be induced after 3 h of α-MSH treatment, following which the MITF protein level decreased with time [34]. A-alum-1 decreased the slightly induced MITF mRNA and protein expression levels after 24 h of α-MSH treatment (Supplementary Figure S1), suggesting that A-alum-1 decreases levels of tyrosinase mRNA and protein in the α-MSH-induced B16F1 melanoma cells via suppressing intracellular MITF mRNA and protein levels. Therefore, we selected 2 and 4 h of α-MSH treatment to assess the effects of A-alum-1 on the expression levels of MITF mRNA and protein, respectively, in α-MSH-induced B16F1 cells. First, we verified the induced expression of MITF mRNA and protein after 2 and 4 h of α-MSH treatment. As shown in Figure 4D, when B16F1 cells were treated with α-MSH for 2 h, the MITF mRNA level significantly increased (~5.57-fold) compared to that observed in the absence of α-MSH (control). A-alum-1 suppressed α-MSH-induced MITF mRNA expression, thereby declining to 49% of the induced MITF mRNA level (Figure 4D). Also, we found that A-alum-1 was able to modestly suppress the α-MSH-induced mRNA expression of MITF and tyrosinase in human melanoma SK-mel-28 cells (Figure 4E), similarly to that in B16F1 cells (Figure 4A,D), suggesting that A-alum-1 can inhibit melanogenesis through a transcriptional suppression-mediated mechanism. Contrary to the marked decrease in the MITF mRNA level by A-alum-1 in B16F1 cells, the α-MSH-induced MITF protein level was only slightly reduced by A-alum-1 after 4 h of α-MSH treatment (Figure 4F). These results indicate that A-alum-1 may downregulate MITF expression mainly at the transcriptional level and partly at the MITF protein level, leading to a decrease in α-MSH-induced tyrosinase gene expression.

### 2.4. A-Alum-1 Decreases α-MSH-Induced MITF Expression via Inhibiting CREB Phosphorylation in B16F1 Cells

CREB and β-catenin are the most well-known coactivators associated with the activation of MITF gene transcription [17,35]. The phosphorylated CREB or accumulated β-catenin stimulates MITF transcription. To further understand the detailed molecular mechanisms involved in the downregulation of MITF transcription by A-alum-1, we first examined whether A-alum-1 affected CREB-mediated MITF transcription in α-MSH-induced B16F1 cells. As shown in Figure 4G, the phosphorylation levels of CREB were enhanced for 30 min, immediately after α-MSH treatment. However, the level of phosphorylated CREB rapidly decreased 1 h after α-MSH treatment. A-alum-1 reduced α-MSH-induced CREB phosphorylation following α-MSH treatment. These results suggested that A-alum-1 suppress MITF transcription by downregulating CREB phosphorylation in B16F1 melanoma cells.

Another upstream coactivator targeting the gene expression of MITF, β-catenin mRNA, and its protein were rarely influenced by treatment with α-MSH and/or A-alum-1 (Figure 4D,F). These data indicate that the β-catenin-mediated transcriptional regulation of MITF is not associated with the inhibition of α-MSH-induced melanogenesis by A-alum-1 in B16F1 cells. Collectively, these results suggested that A-alum-1 attenuated CREB phosphorylation, MITF transcription, and tyrosinase transcription, thereby inhibiting melanogenesis in B16F1 cells. 

### 2.5. A-Alum-1 Inhibits Nuclear Localization of MITF in α-MSH-Induced Melanoma Cells

**Figure 5:**
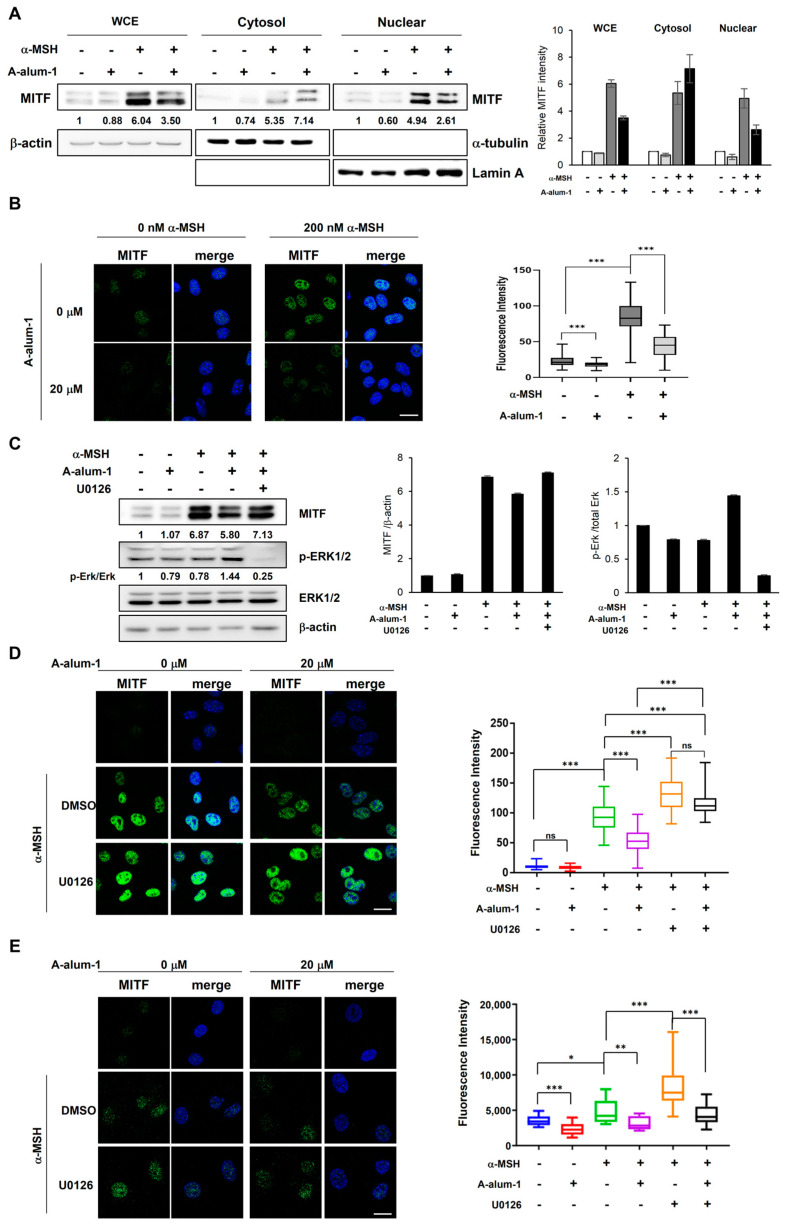
A-alum-1 suppresses the nuclear accumulation of MITF proteins via Erk1/2 signal pathway. B16F1 cells exposed to 200 nM α-MSH in the absence or presence of 20 µM A-alum-1 for 4 h (**A**,**B**). Ten µM U0126, an inhibitor of MEK/Erk1/2, was pretreated for 30 min prior to treatment with A-alum-1 and α-MSH (**C**,**D**). (**A**) Cytoplasmic and nuclear proteins were separated from whole cell extract (WCE) via cellular fractionation. The subcellular distribution of MITF was determined using Western blotting. α-tubulin and lamin A were used as makers for the cytoplasmic and nuclear fractions, respectively. Western blot was representative of three different experiments. Relative protein intensities are shown at the bottom of the Western blot and in the histogram. (**B**) The subcellular localization of MITF was monitored using immunofluorescence. Images were obtained using confocal microscopy; scale bar: 20 μm (400× magnification). Fluorescent intensities of nuclear MITF proteins are presented in the right plot as the means ± SD from at least four experiments. More than 100 cells were counted in each experiment. *** *p* < 0.001 (**C**) The levels of MITF protein and phosphorylation of Erk1/2 were evaluated using Western blotting. Western blot was representative of three different experiments. Relative protein amounts are shown at the bottom of Western blot and in the histogram. The experiment shown in (**B**) was repeated in the B16F1 (**D**) and SK-Mel-28 (**E**) cells pretreated with 10 µM U0126 or not (DMSO). * *p* < 0.05; ** *p* < 0.01; *** *p* < 0.001; ns, not significant.

MITF, a key transcriptional activator of melanogenesis, is located in the nucleus and stimulates the expression of melanogenesis-related genes, leading to melanogenesis. To further investigate whether A-alum-1 influences the nuclear localization of MITF, we first conducted subcellular fractionation and then examined the subcellular distribution of MITF proteins using Western blot analysis. As shown in Figure 5A, the MITF protein elevated by α-MSH was largely located in the nucleus; however, the α-MSH-induced nuclear enrichment of MITF was reduced by A-alum-1 in B16F1 cells. 

To verify the inhibition of the α-MSH-induced MITF nuclear localization by A-alum-1, we next analyzed the MITF subcellular localization using immunofluorescence staining. As shown in Figure 5B, when B16F1 cells were treated with α-MSH, the level of MITF protein was significantly elevated and the MITF protein was sequentially accumulated in the nucleus compared to that observed in the absence of α-MSH (approximately 3.72-fold higher than control), indicating that in the presence of α-MSH, MITF protein levels are significantly increased and the increased MITF translocates to and accumulates in the nuclei. However, A-alum-1 suppressed the nuclear enrichment of MITF by 52% (1.94-fold of control). Taken together, these data suggest that A-alum-1 negatively modulates the subcellular localization of the important melanogenesis transcription factor MITF in B16F1 and SK-Mel-28 melanoma cells, possibly resulting in the downregulation of MITF transcriptional activity for expressing MITF target genes, such as tyrosinase.

### 2.6. A-Alum-1 Inhibits the Nuclear Localization of MITF through Activation of Erk1/2 Signal Pathway in α-MSH-Induced Melanoma Cells

The activation of ERK inhibits melanin synthesis through the nuclear export and cytoplasmic accumulation of MITF proteins [36]. To further understand the mechanisms underlying MITF downregulation by A-alum-1, we investigated whether the inhibition of MITF nuclear localization by A-alum-1 involves ERK activation in two melanoma cell lines, B16F1 and SK-Mel-28. The level of phosphorylated ERK was increased by A-alum-1 in α-MSH-induced B16F1 cells (Figure 5C and Supplementary Figure S2), suggesting that A-alum-1 can activate ERK in α-MSH-induced cells. As shown in Figure 5D,E, the ERK inhibitor U0126 completely inhibited ERK phosphorylation (Figure 5C), strengthened MITF nuclear accumulation, and abrogated the inhibitory effects of A-alum-1 on MITF nuclear accumulation (Figure 5D,E). These results verify that the activation of the ERK pathway inhibits the nuclear localization of MITF, indicating that A-alum-1 inhibits MITF nuclear accumulation by activating ERK signaling in B16F1 and SK-Mel-28 melanoma cells.

## 3. Discussion

**Figure 6:**
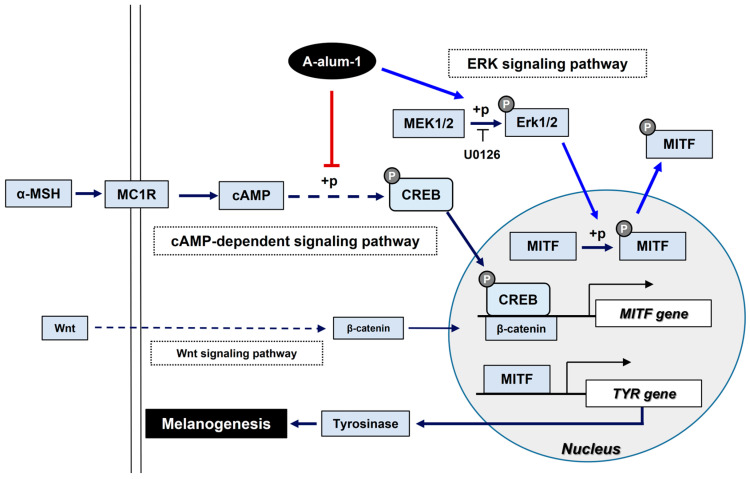
Schematic diagram presenting the proposed anti-melanogenic activity of A-alum-1 in B16F1 cells. A-alum-1 modulates MITF, the melanogenic transcription factor, at both transcriptional and post-translational levels via the downregulation of p-CREB and activation of Erk1/2 signal pathway, respectively. The inhibition of CREB phosphorylation by A-alum-1 corresponds to decreased MITF expression, ultimately leading to reduced tyrosinase expression. Erk1/2 activation contributes to a dramatic decrease in the nuclear localization of the MITF protein, which consequently inhibits melanogenesis. These modulations of melanogenesis-related proteins by A-alum-1 eventually result in decreased melanin contents in A-alum-1-treated melanoma cells.

Alum, a compound containing aluminum, usually refers to potassium alum, a mineral salt. Owing to its astringent and antibacterial properties, it has various cosmetic and medical uses, including in styptic products, deodorants, face tonics, preservatives, anti-acne products, and vaccine adjuvants. In addition, alum is often an ingredient in skin-whitening products; however, its efficacy and the molecular mechanisms underpinning its skin-lightening effects have been largely overlooked. Moreover, to the best of our knowledge, no study has compared the efficacy and/or safety of anhydrous and hydrated alums. Paradoxically, this oversight has been in part due to its widespread use for quite a long time. Here, we report that A-alum-1 may be a safe (noncytotoxic) and potent skin-whitening ingredient. This assertion is based on our findings that A-alum-1 displayed anti-melanogenic effects via the downregulation of the key melanogenesis MITF transcription factor through multiple pathways, including the cAMP-CREB and ERK pathways in B16F1 cells (Figure 6).

Despite its likely reliable efficacy, we found little anecdotal evidence for the skin-lightening effects of alum. We used anhydrous alum with high purity (A-alum-1) via roasting the hydrated alum at 400 °C to elucidate its melanogenesis inhibitory effects in melanoma B16F1, SK-Mel-28 and G-361 cells and molecular mechanisms in B16F1 cells (Figure 1 and Figure 2). Heat treatment at a higher temperature (500 °C) afforded impure anhydrous alum containing aluminum sulfate as the impurity phase. In the present study, A-alum-1 was a more effective anti-melanogenic substance than hydrated or commercial anhydrous alum (Figure 3), implying that its structural features and purity (Figure 1 and Figure 2) play an important role in the downregulation of MITF, thereby inhibiting melanogenesis. However, in the present study, the inhibition of melanogenesis by A-alum-1 and the underlying mechanisms were investigated at the cellular level in α-MSH-induced B16F1 cells. Therefore, further investigations across normal melanocytes together with mechanism studies still need to be performed to ensure the anti-melanogenic effects of A-alum-1 and to better understand its action mechanism in response to a variety of signals.

Tyrosinase is the rate-limiting enzyme in the sequence of reactions and controls intracellular melanin production. Therefore, increased tyrosinase levels correlate with increased melanin production. A-alum-1 inhibited melanin production in a dose-dependent manner in α-MSH-induced melanoma cells (Figure 3C,D), alongside the suppression of tyrosinase expression and activity (Figure 4A–C).

Our findings may further establish a mechanistic basis for the dual melanogenesis regulatory pathways via the downregulation of the master melanogenesis regulator MITF by A-alum-1, which acts upstream of tyrosinase, in B16F1 cells (Figure 4D,F, Figure 5 and Figure 6). MITF is a transcriptional activator responsible for the expression of melanogenic enzymes including tyrosinase (Figure 6) [12]. Accordingly, the upregulation of MITF correlates with the enhanced expression of melanogenesis enzymes, thereby stimulating melanogenesis [4,18]. Conversely, the downregulation of MITF suppresses transcription, thereby inhibiting melanin production [34,37].

α-MSH secreted in response to UV light activates the cAMP-PKA pathway, which elevates CREB phosphorylation, ultimately resulting in the upregulation of MITF gene expression [37,38]. In agreement with previous results, upon the stimulation of melanocytes with α-MSH, the cAMP-PKA pathway enhanced CREB phosphorylation, which in turn assisted the transcription of the MITF gene as a coactivator, leading to increased MITF mRNA levels. Indeed, α-MSH significantly induced CREB phosphorylation and MITF mRNA expression in B16F1 cells (Figure 4D), supporting the idea that the activated cAMP-PKA pathway induces MITF mRNA expression in α-MSH-stimulated B16F1 cells. In this pathway, A-alum-1 was competent to rapidly inhibit CREB activation by interfering with CREB phosphorylation, thereby attenuating the α-MSH-induced MITF transcription (Figure 4D). These results suggest that A-alum-1 downregulates MITF gene expression in α-MSH-induced B16F1 melanoma cells. 

In addition to downregulating MITF gene expression, A-alum-1 activated ERK phosphorylation, which disrupted the nuclear localization of MITF in B16F1 and SK-Mel-28 cells (Figure 5 and Figure 6). We confirmed that A-alum-1 significantly suppressed MITF nuclear accumulation in α-MSH-treated cells via ERK activation using an ERK inhibitor, which abolished the A-alum-1-mediated dramatic reduction in nuclear MITF levels in melanoma cells (Figure 5C–E). Hence, these findings suggest that the activation of ERK by A-alum-1 also suppresses α-MSH-induced melanogenesis by interrupting MITF nuclear accumulation in melanoma cells.

In contrast to the downregulation of MITF by A-alum-1 via engaging in the cAMP-PKA and ERK pathways, A-alum-1 did not influence the β-catenin-TCF/LEF pathway (Figure 4F and Figure 6). Furthermore, A-alum-1 did not affect the stress-activated kinases JNK and p38, which regulate melanogenesis [39], in contrast to ERK activation, in B16F1 cells (Supplementary Figure S2). 

Yet, this work using melanoma cells calls for further investigations using normal melanocytes and animals because the differences in the molecular mechanisms and metabolism of normal and cancer cells, as well as gene mutations in cancer cells, may differentially affect melanogenesis in normal melanocytes and melanoma cells. Moreover, the repetitive use of alum might induce its accumulation in skin cells or the body, possibly leading to its side effects or aluminum poisoning. Therefore, further studies are required to better understand its accumulation process for side-effect prevention and detoxification. 

In conclusion, the present study demonstrates that high-purity anhydrous alum (A-alum-1) inhibits melanogenesis via the cAMP-PKA/CREB pathway-related downregulation of MITF gene expression (at the transcriptional level) and ERK pathway-related impairment in MITF nuclear accumulation (at the post-translational level) in α-MSH-induced melanoma cells (Figure 6). Although our present results highlight the anti-melanogenic effect of A-alum-1 on several melanoma cell lines and the underlying molecular mechanisms in B16F1 cells, the majority of the experiments for the mechanism of its action were limited to one melanoma B16F1 cell line. Thus, further studies using normal melanocytes and in vivo systems are required for validating its effects and action mechanisms, and, further, its application.

## 4. Materials and Methods

### 4.1. Preparation and Characterization of Anhydrous Alum

Hydrated potassium alum (99.9%), KAl(SO_4_)_2_·12H_2_O (H-alum), was purchased from Samhyun Pharm (Incheon, Republic of Korea). Anhydrous potassium alum, KAl(SO_4_)_2_ (denoted as A-alum-1), was synthesized via the dehydration of H-alum at 400 °C for 4 h. For comparison, a commercial roasted potassium alum (denoted by A-alum-2) was purchased from Hyunjin Pharm (Seoul, Republic of Korea), which is typically produced by the heating of hydrated alum at 500 °C. 

XRD measurements were performed using a Rigaku D/MAX2200PC with Cu Kα radiation (λ = 1.5418 Å). The data were recorded at room temperature in the 2θ range of 5–90° with a step size of 0.02° and a counting time of 4 s per step. Thermogravimetric analyses were performed using an STA 409 PC thermal analyzer (NETZSCH, Aacheen, Germany). Approximately 20 mg of sample was weighed in an alumina crucible, followed by heating from 25 °C to 500 °C under air condition at a heating rate of 10 °C/min. A Scanning Electron Microscope (SEM) was used to image the samples, employing JSM-6380 (JEOL, Akishima, Japan). 

### 4.2. Cells and Reagents

B16F1 cells (80007), derived from murine melanoma cells, were obtained from the Korean Cell Line Bank (KCLB, Seoul, Republic of Korea) and HaCaT (human keratinocyte), G-361, and SK-Mel-28 (human melanoma cell lines) were obtained from the American Type Culture Collection (ATCC). B16F1 and HaCaT cells were maintained in Dulbecco’s Modified Eagle medium (DMEM) (Welgene Inc., Gyeongsan, Republic of Korea) supplemented with 10% fetal bovine serum (FBS) (Hyclone, GE Healthcare, Chicago, IL, USA) and 100 U/mL penicillin/streptomycin (Welgene Inc., Gyeongsan, Republic of Korea). G-361 cells were cultured in RPMI-1640 containing 10% FBS and antibiotics. SK-Mel-28 cells were cultured in Eagle’s Minimum Essential Medium (EMEM) containing 10% FBS and antibiotics. The cells were cultured in a humidified incubator containing in 5% CO_2_ at 37°C. Potassium aluminum sulfate was obtained from Sigma-Aldrich (St. Louis, MO, USA) and U0126 was purchased from Enzo Life Sciences (Farmingdale, NY, USA).

### 4.3. Cell Viability Assay

B16F1 and HaCaT cells (1 × 10^4^ cells/well) were plated in 96-well plates and allowed to attach for 24 h before treatment. The cells were treated with various concentrations of A-alum-1, A-alum-2, and H-Alum for 48 h prior to the cell viability assay. Cell viability was measured using the resazurin assay [40]. Resazurin solution was added to a final concentration of 100 μM, followed by incubation for 3 h. Resazurin reduction was determined by measuring the absorbance at 595 nm using an Epoch Microplate Spectrophotometer (BioTek Instruments, Inc., Winooski, VT, USA). All experiments were conducted in triplicates.

### 4.4. Melanin Content and Tyrosinase Activity Assays

Melanin content was assessed using a previously described spectrometric quantification method [41] with slight modifications. Briefly, B16F1, G-361, and SK-Mel-28 cells (5 × 10^4^ cells/mL) were plated in 6-well plates and allowed to attach for 24 h. The cells were then treated with or without A-alum-1, H-alum, or A-alum-2 (0, 10, or 20 μM) 1 h ahead of a further 72 h in the presence or absence of 200 nM α-MSH. After incubation, these cells were lysed with 0.1 N NaOH for 1 h at 90 °C to harvest clear lysates. The amount of melanin in the lysates was monitored by measuring absorbance at 450 nm. Tyrosinase activity was determined using a previously described cell-based colorimetric assay [42]. B16F1 cells (6 × 10^5^ cells) were plated in a 60 mm culture dish and allowed to attach for 24 h. The cells were pretreated with 0 or 20 μM of A-alum-1 for 1 h and then further incubated for 72 h in the presence of 200 nM α-MSH. After incubation, cells were lysed with a 0.1 M phosphate buffer (pH 6.8) containing 1% Triton X-100 and a protease inhibitor cocktail (Roche, Basel, Switzerland). The supernatants were measured to determine the protein concentration using the Lowry assay system. For the DOPA oxidase activity of tyrosinase, each sample was incubated with 2 mM L-DOPA (Sigma) in a 0.1 M phosphate buffer (pH 6.8) for 90 min at 37 °C. After incubation, the DOPA oxidase activity was measured at 490 nm with an ELISA reader (Bio-Rad, Hercules, CA, USA). Each treatment was performed in triplicate and each experiment was repeated six times. 

### 4.5. RNA Isolation and Quantitative Reverse Transcription PCR (qRT-PCR)

Total RNA was isolated using the Hybrid-R Total RNA Purification Kit (GeneAll, Seoul, Republic of Korea). One μg of total RNA was reverse-transcribed with PrimeScript RT Master Mix (Takara Bio, Shiga, Japan). After a 1:10 dilution, 2 μL of cDNA was used as a template in a 20 μL PCR mixture. The following primers were used for qPCR: mouse TYR (5′-TTGCCACTTCATGTCATCATAGAATATT-3′ and 5′-TTTATCAAAGGTGTGAC- TGCTATACAAAT-3′); mouse MITF (5′-CGCCTGATCTGGTGAATCG-3′ and 5′-CCTGG- CTGCAGTTCTCAAGAA-3′); mouse CTNNB1 (5′-GACACCTCCCAAGTCCTTTATG-3′ and 5′- CTGAGCCCTAG TCATTGCATAC-3′); mouse GAPDH (5′-ACTCCACTCACGGCAAATTC-3′ and 5′-TCTCCATGGTGGTGAAGACA-3′); mouse RPL13 (5′-GGCCAGAGTTATCACAGAAGAA-3′ and 5′-TTGCTC GGATGCCAAAGA-3′); human TYR (5’-TCAT- CCAAAGATCTGGGCTATGACT-3’ and 5’-GTGACGACACAGCAAGCTCAC-3’); human MITF (5’-GTGTCACTGATCCACTCCTTTC-3’ and 5’-CCGTCTCTTCCATGCTCAT- AC-3’); human CTNNB1 (5’-CTTCACCTGACAGATCCAAGTC-3’ and 5’-CCTTCCATC- CCTTCCTGTTTAG-3’); human GAPDH (5’-TGAAGCAGGCATCTGAGGG-3’ and 5’-C- GAAGGTGGAAGAGTGGGAG-3’).

The relative quantification of gene expression was carried out using the ΔΔCt method with GAPDH and RPL13 as reference genes. The PCR program was as follows: initial denaturation at 95 °C for 2 min, followed by 45 cycles of 95 °C for 5 s, and 60 °C for 30 s. The final amplification cycle was followed by a melting curve analysis to confirm the specificity of the PCR amplification.

### 4.6. Western Blot Analysis

The cells were lysed in radioimmunoprecipitation assay (RIPA) buffer [50 mM Tris-Cl (pH8.0), 150 mM NaCl, 0.1% SDS, 0.5% deoxycholic acid, 1% NP-40] containing a protease inhibitor and a phosphatase inhibitor cocktail and briefly sonicated. Protein concentration was determined using the Pro-Measure^TM^ Protein Measurement solution (iNtRON Biotechnology, Seongnam, Republic of Korea). The cell lysates containing equal protein amounts (5–20 µg) were subjected to 10 or 12% SDS polyacrylamide gel electrophoresis (SDS-PAGE). After electrophoresis, the separated proteins were transferred onto nitrocellulose membranes (Cytiva Life Sciences, Buckinghamshire, UK) using a transfer system (Bio-Rad Laboratories, Hercules, CA, USA). Membrane blocking was performed with 5% skim milk for 1 h, and followed incubation with the primary antibodies at 4 °C overnight. The following primary antibodies were purchased: tyrosinase, β-catenin, β-actin (Santa Cruz Biotechnology, Santa Cruz, CA, USA), MITF, phospho-CREB, CREB, phospho-Erk1/2, Erk1/2 (Cell Signaling Technology, Danvers, MA, USA), α-tubulin (Abcam, Cambridge, UK), and Lamin A (Sigma-Aldrich). Horseradish peroxidase (HRP)-conjugated secondary antibodies were purchased from Enzo Life Sciences. Targeted proteins were detected using enhanced chemiluminescence (ECL) Western blotting detection reagents (DONGIN LS Co., Ltd., Hwaseong, Republic of Korea). The Western blot results reported here are representative of at least three experiments. 

### 4.7. Nuclear and Cytoplasm Subcellular Fractionation

To isolate subcellular fractions, the cells were suspended in 400 μL of Buffer A [10 mM HEPES-KOH (pH 7.5), 1.5 mM MgCl_2_, 10 mM KCl, 0.5 mM DTT, 0.5 mM PMSF] supplemented with protease inhibitor and phosphatase inhibitor cocktails for 15 min on ice and with 8 μl of 10% NP-40 (final 0.2%) added, followed by vigorous vortex mixing for 10 s. Cellular suspensions were centrifuged at 14,000 rpm for 5 min at 4 °C, and the supernatant was transferred to fresh tubes (cytoplasmic fraction). The pellets were resuspended in 200 μl of RIPA buffer and sonicated (nuclear fraction). Lamin A and α-tubulin were used as markers for the nuclear and cytoplasmic fractions, respectively.

### 4.8. Immunofluorescence 

B16F1 and SK-Mel-28 cells (2 × 10^5^ cells/well) were plated in 12-well plates and allowed to attach for 24 h. After treatment with or without A-alum-1 (20 μM) and α-MSH (200 nM), cells were fixed with 4% paraformaldehyde (10 min at 25 °C). The cells were washed with 1× PBS containing 0.1% Tween 20 (PBST) thrice. Fixed cells were permeabilized with 0.5% Triton X-100 for 10 min. After washing, the cells were blocked with 1% BSA in PBST for 1 h and incubated with MITF antibody in antibody dilution buffer [1× PBS, 1% BSA, 0.1% Triton X-100, 0.1% NaN_3_] for 1~2 h at 37 °C or overnight at 4 °C. After washing, the cells were incubated with Alexa 488-conjuated secondary antibody (Invitrogen) for 1 h at room temperature. After counterstaining with 4′6-diamidino-2-phenylindole (DAPI) for nuclei, immunofluorescence images were captured with a laser-scanning confocal microscope (LSM800; Carl Zeiss Microimaging, Jena, Germany). 

### 4.9. Statistical Analysis

All data were obtained in triplicate and are presented as the means ± standard deviation. Values were compared using the Student’s *t*-test and one-way analysis of variance (ANOVA). Statistical significance was set at *p* < 0.05. Significance degrees are indicated as follows: * *p* < 0.05 and ** *p* < 0.01, *** *p* < 0.001.

## Data Availability

Data are available from the corresponding authors upon reasonable request.

## Supplementary Materials

The following supporting information can be downloaded at https://www.mdpi.com/article/10.3390/ijms241914662/s1.

## Abbreviations

MITF: microphthalmia-associated transcription factor; CREB: cAMP response element-binding protein; TYR: tyrosinase; α-MSH: α-melanocyte-stimulating hormone; ERK: extracellular signal-regulated kinase; qRT-PCR: quantitative reverse transcription-polymerase chain reaction
