# Supplementary material for: Anhydrous Alum Inhibits α-MSH-Induced Melanogenesis by Down-Regulating MITF via Dual Modulation of CREB and ERK
Source: Int J Mol Sci. 2023 Sep 28;24(19):14662. doi: 10.3390/ijms241914662 (PMC10572554; doi:10.3390/ijms241914662)
Supplement: Supplementary file 1 [file ijms-24-14662-s001.zip › Supplementary Figure Legends (V4).pdf]

Supplementary Figure Legends

Supplementary Figure S1. Effect of A-alum-1 on the mRNA and protein levels of MITF and β-catenin. B16F1 cells were exposed to 200 nM α-MSH in the presence of 0 or 20 μM A-alum-1. After 24 h incubation, the expression levels of MITF, β-catenin and tyrosinase were measured using qRT-PCR (A) and western blot analysis (B). β-actin used as a loading control.

Supplementary Figure S2. Effects of A-alum-1 on the MAPK signaling pathway. B16F1 cells were exposed to 200 nM α-MSH in the presence of 0 or 20 μM A-alum-1. The phosphorylation levels of Erk1/2, JNK1/2 and p38 was determined by western blot analysis
