# Supplementary figures and images for: Anhydrous Alum Inhibits α-MSH-Induced Melanogenesis by Down-Regulating MITF via Dual Modulation of CREB and ERK
Source: Int J Mol Sci. 2023 Sep 28;24(19):14662. doi: 10.3390/ijms241914662 (PMC10572554; doi:10.3390/ijms241914662)

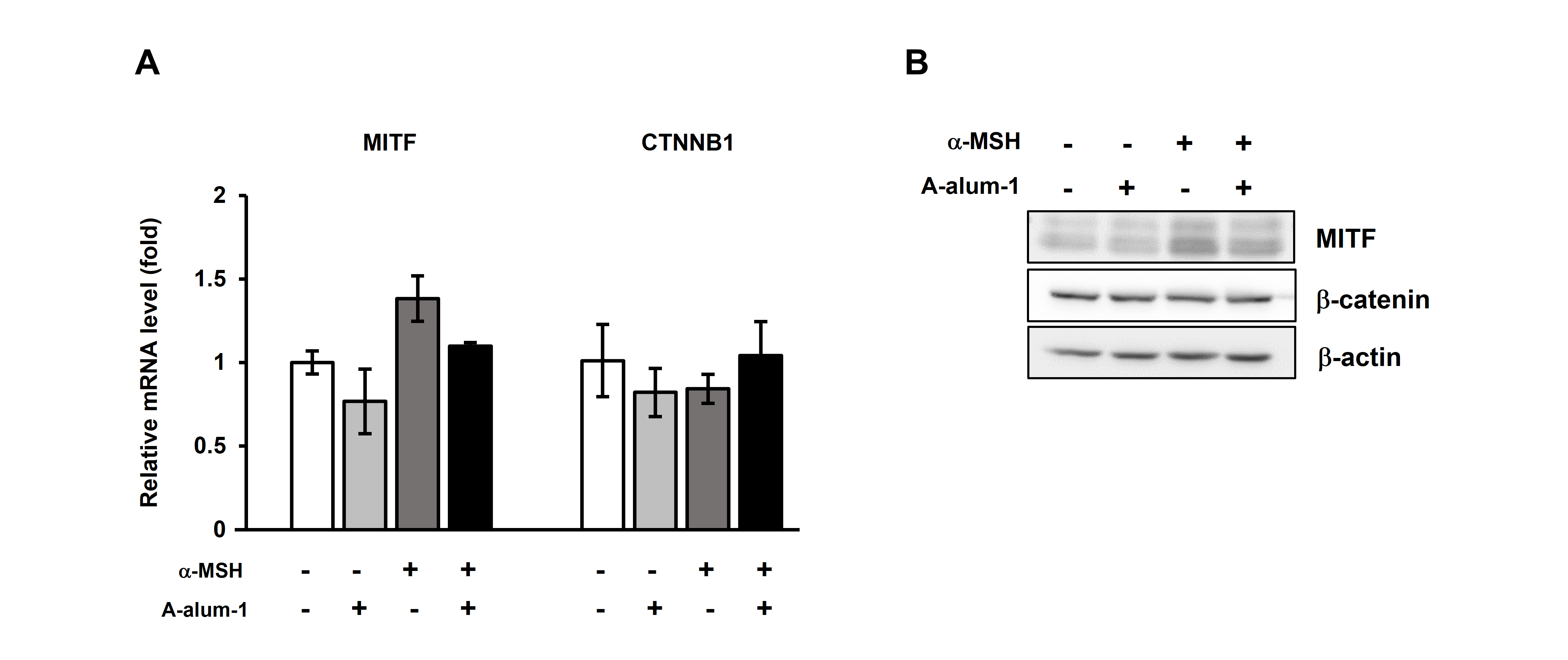

Supplement: Supplementary file 1 [file ijms-24-14662-s001.zip › Supple 1.tif]

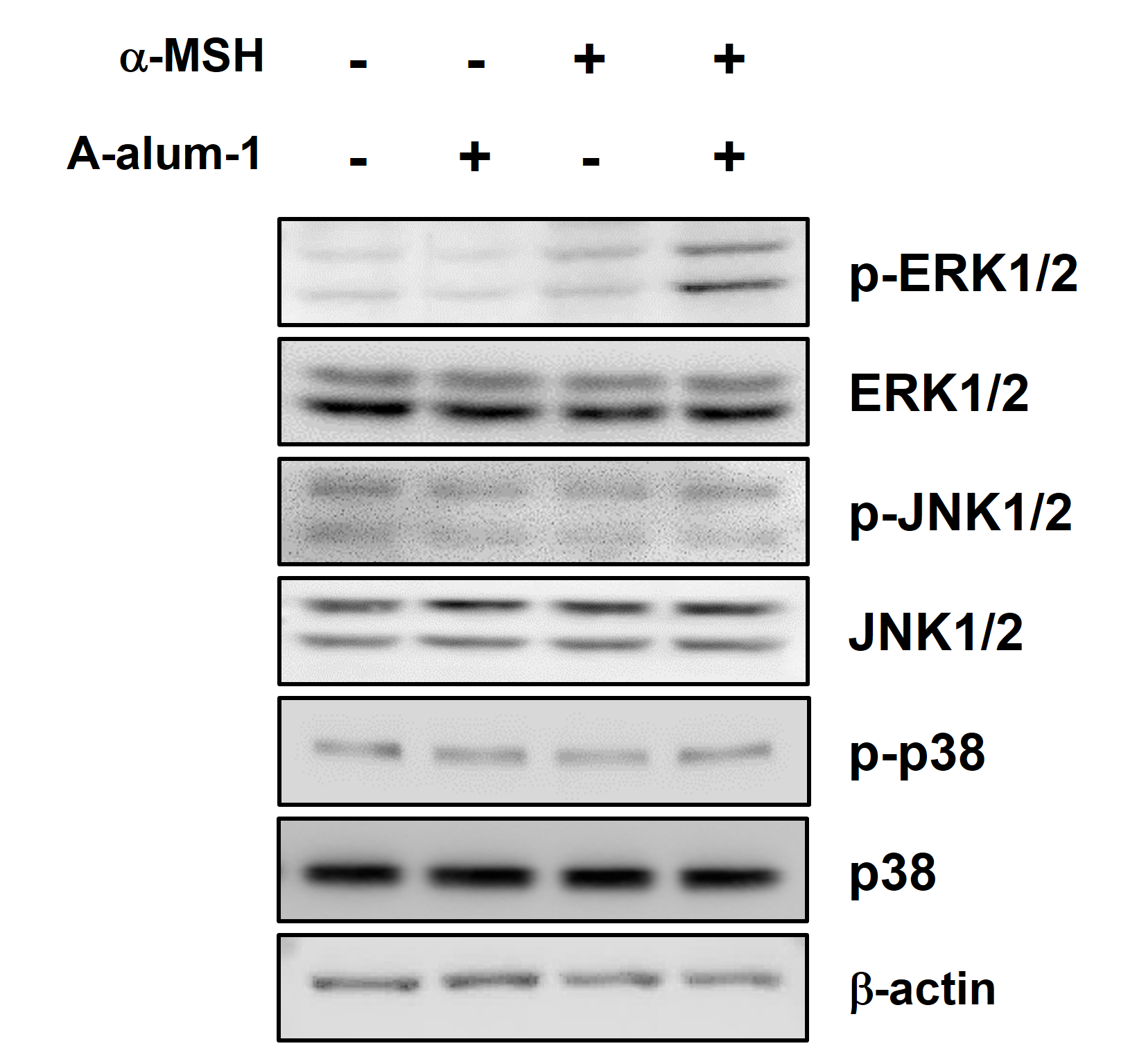

Supplement: Supplementary file 1 [file ijms-24-14662-s001.zip › Supple 2.TIF]
